# Identifying characteristics of indicators of sedentary behavior using objective measurements

**DOI:** 10.1002/1348-9585.12089

**Published:** 2019-10-10

**Authors:** Kojiro Yamamoto, Fumiko Matsuda, Tsuyoshi Matsukawa, Nao Yamamoto, Kenji Ishii, Takahiro Kurihara, Shota Yamada, Taro Matsuki, Michihiro Kamijima, Takeshi Ebara

**Affiliations:** ^1^ Nagoya City University Graduate School of Medical Sciences Nagoya Japan; ^2^ The Ohara Memorial Institute for Science of Labour Tokyo Japan; ^3^ Faculty of Information Science Aichi Institute of Technology Toyota Japan; ^4^ Nagoya City University Graduate School of Economics Nagoya Japan

**Keywords:** obesity, objective measurement, sedentary behavior, smartphone

## Abstract

**Objective:**

Recent attention has been focused on sedentary behavior (SB) affecting health outcomes, but the characteristics of indicators reflecting SB remain to be identified. This cross‐sectional study aims to identify the characteristics of indicators of SB, focusing on the examination of correlations, reliability, and validity of sedentary variables assessed by the smartphone app.

**Method:**

Objectively measured data of SB of eligible 46 Japanese workers obtained from smartphones were used. We assessed the characteristics of current indicators being used with a 10‐minute or 30‐minute thresholds, in addition to the conventional indicators of total sedentary time, mean sedentary bout duration, and total number of sedentary bouts. They were evaluated from three perspectives: (a) association among the indicators, (b) reliability of the indicators, and (c) criterion validity.

**Results:**

*Total sedentary time under 10 minutes* (*U10*) and *U30* had negative associations with *Total sedentary time* (r = −.47 and −.21 respectively). The correlation between *Mean sedentary bout duration* and *Total number of sedentary bouts* was −.84, whereas between *Mean sedentary bout duration 10*, *30* and *Total number of sedentary bouts* were −.54 and −.21, respectively. The intraclass correlation coefficients of almost all indicators were around .80. *Mean sedentary bout duration*, *Mean sedentary bout duration 10*, *Total number of sedentary bouts*, *Total sedentary time 30*, *U30* and *U10* have significant differences between three BMI groups.

**Conclusion:**

This study comprehensively revealed the rationale of advantage in the current indicator being used with a 10‐minute or 30‐minute threshold, rather than the conventional total amount of SB.

## 1 INTRODUCTION

In the past decade, there has been increasing evidence showing that high amounts of sedentary behavior (SB) such as sitting or reclining increase the risk of noncommunicable disease and mortality independent of physical activity.^1^, ^2^, ^3^, ^4^, ^5^ Although objective measurement has been used to accurately measure sedentary time in recent years, associations between SB and body mass index (BMI) as a surrogate health outcome of the noncommunicable disease are varied and contentious among the studies.^6^, ^7^, ^8^, ^9^ A prospective study using an accelerometer reported that significant associations were observed between prolonged sedentary time (sedentary bout duration ≥30 minutes) and metabolic syndrome, whereas total sedentary time was not associated with the syndrome.^10^ Cross‐sectional studies reported that an increase in the number of sedentary bouts showing sit‐to‐stand transitions was associated with a decrease in waist circumference.^2^, ^11^, ^12^ However, some other studies reported no associations between the number of sedentary breaks and waist circumference.^13^, ^14^ One of the reasons for this inconsistency can be found in the objective indicators used in the studies; that is, how to define SB and calculate indicators has been little considered. A previous study, for instance, provided notable evidence that a minimum sedentary bout duration of 1 minute defined as sedentary time underestimated the risk of metabolic syndrome and diabetes compared with a minimum sedentary bout duration of 10 minutes.^15^ Thus, attention has been focused on the indicators being used with a 10‐minute or 30‐minute thresholds, rather than the amount of SB, but the characteristics of such indicators remain to be identified.

Smartphones have various sensors that can be used to measure daily physical activity, including an accelerometer or gyroscope. Recent study revealed the moderate to strong correlations of physical activity level estimates between the ActiGraph (well‐used activity monitoring accelerometer device) and general smartphones in free‐living settings.^16^ Smartphone devices could provide enough information to estimate sedentary time in daily life though, much evidence on its' availability for academic research would be needed. Thus, smartphones are now paid attention as a potentially cost‐efficient and low‐burden way for tracking daily physical activity.^16^, ^17^, ^18^, ^19^The aim of this cross‐sectional study, therefore, was to identify the characteristics of indicators of SB, focusing on the examination of correlations, reliability, and validity of sedentary variables assessed by the smartphone app.

## 2 METHODS

### 2.1 Survey data

**Figure 1:**
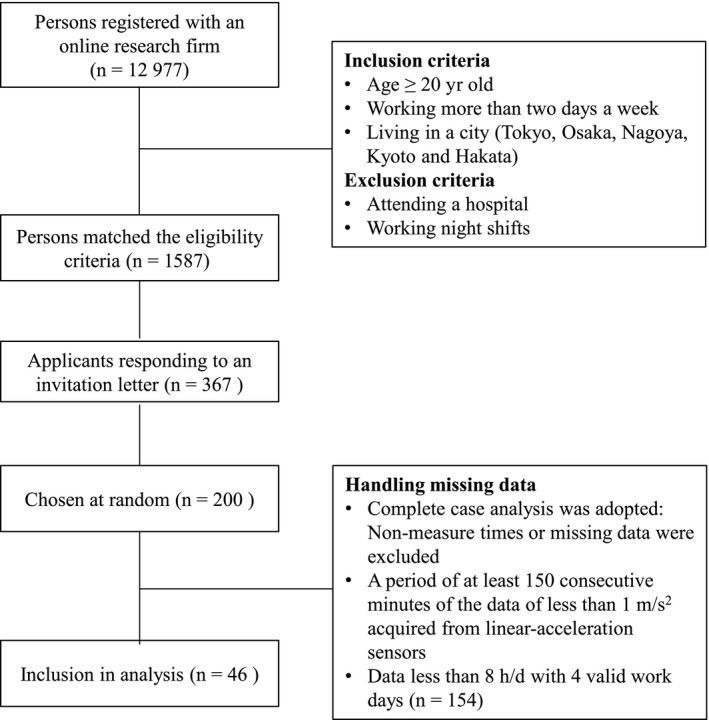
Participant selection diagram

The participants were recruited from individuals registered as monitors with an online research firm. For inclusion, participants had to be aged 20 years or older, work more than 2 days a week, and be living in a city (Tokyo, Osaka, Nagoya, Kyoto, and Hakata). Those who attended a hospital and worked night shifts were excluded. The online research firm was responsible for recruiting participants, sending and collecting documents (detailed study overview and consent form), and responding to enquiries. A total of 1587 of the 12 977 people who matched the criteria applied for this study. The firm sent an invitation letter to them, and 367 applicants responded. Of these, 200 applicants were chosen at random (Figure 1). Prior to participating in the survey, they were asked to install a smartphone app for academic research, Motion Logger Ver. 1.5, and to place their phone in a chest pocket for lifelog measurement over 7 consecutive days. They completed a basic questionnaire (eg, age, sex, height, and weight, etc) on the first day and an additional questionnaire (eg, sleeping time and waking time, etc) every morning and evening on a smartphone. Written informed consent for the research was obtained from all participants before the survey began. This study was approved by the Institutional Review Board of Nagoya City University Graduate School of Medical Sciences (No. 60 160 123).

### 2.2 App specification

The app, Motion Logger Ver. 1.5, measures time‐series log data using 10 key indicators obtained from embedded hardware sensors in smartphones, such as a step‐counting sensor. The information can be measured for 7 consecutive days at a sampling rate of 500 ms. The reliability of the data obtained from the app was confirmed by the test of the false detection rate during various sitting situations.^20^

### 2.3 Conventional and current indicators of sedentary behavior

Studies on SB often tend to use the following conventional indicators.^5^, ^21^, ^22^, ^23^, ^24^

***Total sedentary time*** *(h/d)*: the total time of SB in a day

***Mean sedentary bout duration*** *(min/d)*: the mean of sedentary bout duration

***Total number of sedentary bouts*** *(number of times/d)*: the number of occurrences of continuous sedentary time with no interruption

In addition to above, some recent studies operationally define the thresholds of sedentary times or duration as ≥30‐minutes, or ≥10‐minutes to estimate nature of SB.^9^, ^10^, ^15^, ^25^, ^26^, ^27^ Therefore, the current indicators being used were defined as follows:

***Total sedentary time 30*** *(h/d)*: the total time of sedentary bout duration over 30 minutes

***Total sedentary time U30*** *(h/d)*: the total time of sedentary bout duration under 30 minutes

***Total sedentary time 10*** *(h/d)*: the total time of sedentary bout duration over 10 minutes

***Mean sedentary bout duration 30*** *(min/d)*: the mean of sedentary bout duration over 30 minutes

Relevant to the current indicators, we additionally defined secondary ones that were little used as yet in scientific papers as follows:

***Total sedentary time U10*** *(h/d)*: the total time of sedentary bout duration under 10 minutes

***Mean sedentary bout duration 10*** *(min/d)*: the mean of sedentary bout duration over 10 minutes

Since the indicators related to *Total sedentary time* and *Total number of sedentary bouts* were affected by measurement time, these indicators were adjusted to mean measurement time (16.3 hours) using the residuals obtained from linear regression models.^9^, ^28^

### 2.4 Statistical analysis

In this study, data resampled every 5 seconds for at least 8 h/d of participants with more than 4 valid work days (n = 46) were used because there is a previous study reporting that 3‐7 days are needed to achieve a reliable estimate of sedentary behavior and physical activity using an accelerometer. The intraclass correlation coefficients (ICCs) of SB also become higher in case of limiting it to only working hours (except for leisure time) (Figure 1).^29^ Thus, 4 valid work days were extracted according to a descending order of measurement time (person‐days = 184). Non‐measurement times or missing data were excluded. These were identified by comparing the output data with the participants’ self‐reports of daily activities such as hours of sleep. In addition, a period of at least 150 consecutive minutes of data of less than 1 m/s^2^ acquired from linear‐acceleration sensors was also regarded as non‐measurement times.

**Figure 2:**
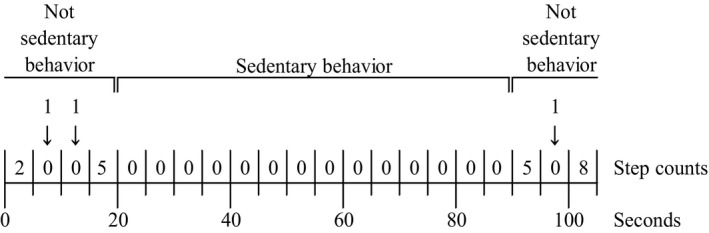
Data processing: A period of at least 60 s of 0 steps, as measured by a step‐counting sensor, is defined as sedentary behavior. A period of less than 60 s of 0 steps is defined as not sedentary behavior by converting 0 steps to 1 step

Sedentary behavior was defined as a period of at least 60 seconds of 0 steps, as measured by a step‐counting sensor. A period of less than 60 seconds of 0 steps was defined as not SB by converting 0 steps to 1 step (Figure 2).

The characteristics of indicators were evaluated from three perspectives: (a) association among the indicators, (b) reliability of the indicators, and (c) criterion validity. To examine the association between indicators, Pearson's correlation coefficient was calculated. It was assumed that sedentary patterns were similar if work styles were not different; therefore, the reliability of the indicators could be evaluated using the intraclass correlation coefficient (ICC_1_) for 4 work days. Indicators with ICC_1_ over 0.8 were defined as reliable.^30^ The participants were divided into three groups by self‐reported BMI (underweight: ≤18.4 kg/m^2^, normal: 18.5‐24.9 kg/m^2^, overweight: ≥25.0 kg/m^2^). Criterion validity was evaluated using the three group differences of mean indicator values of the 4 work days. Analysis of covariance (ANCOVA) adjusted for age, sex, and physical activity was performed. Mets h per week was used to adjust for physical activity according to the participants’ self‐reports based on the International Physical Activity Questionnaire (IPAQ).^31^ Significance was set at *P* < .05. The distribution of the indicators of 184 person‐days (skewness and kurtosis) was also evaluated as characteristics of indicators. Data processing and analyses were conducted using R Version 3.5.0.

## 3 RESULTS

### 3.1 Characteristics of the participants and distributions of indicators

Table 1 shows the characteristics of the participants and the indicators of SB. Of the 46 participants, 22 (47.8%) were men. The mean age of all participants was 38.3 ± 7.8 years and the mean BMI was 22.0 ± 3.6 kg/m^2^. The mean measured time of the valid days was 16.3 ± 2.7 h/d. The conventional indicators, except for *Total number of sedentary bouts*, had large skewness and kurtosis. The indicators except *Mean sedentary bout 30* and *10* had small skewness and kurtosis, which followed the normal distribution.

**Table 1:** Characteristics of participants and indicators

	Descriptive statistics	Distribution of the indicators^a^	
		Skewness	Kurtosis
Demographic characteristics			
Age (y), mean (SD)	38.3 (7.8)		
Sex (male), % (n)	47.8 (22)	—	—
BMI (kg/m^2^), mean (SD)	22.0 (3.6)	—	—
Measurement time (h/d), mean (SD)	16.3 (2.7)	—	—
Conventional indicators, mean (SD)			
Total sedentary time (h/d)	14.2 (1.2)	−1.42	3.71
Mean sedentary bout duration (min/d)	14.0 (7.0)	1.22	1.54
Total number of sedentary bouts (number of times a day)	75.4 (32.0)	**0.80**	**0.50**
Current indicators, mean (SD)			
Total sedentary time 30 (h/d)	7.9 (2.7)	−**0.09**	−**0.52**
Total sedentary time U30 (h/d)	6.3 (2.1)	**0.04**	−**0.57**
Total sedentary time 10 (h/d)	11.2 (2.3)	−**0.65**	**0.08**
Mean sedentary bout duration 30 (min/d)	72.9 (30.6)	3.64	1.58
Secondary indicators, mean (SD)			
Total sedentary time U10 (h/d)	3.0 (1.4)	**0.50**	−**0.43**
Mean sedentary bout duration 10 (min/d)	39.1 (12.8)	1.13	23.7

a The values of skewness and kurtosis near zero can be interpreted as indicating normality of a given data set (184 person‐days) of each indicator. Boldface indicates values of Skewness and Kurtosis < | 1.0 |.

### 3.2 Associations between conventional indicators and current indicators

Table 2 presents the results for the associations between conventional indicators and current indicators. *Total sedentary time U10* and *U30* had negative associations with the conventional indicator of *Total sedentary time* (r = −.47 and −.21, respectively). *Total sedentary time 10* and *30* had positive associations with conventional *Total sedentary time* (r = .83 and r = .63, respectively).

**Table 2:** Correlation coefficients between conventional indicators and current indicators

	Total sedentary time	Mean sedentary bout duration	Total number of sedentary bouts
Conventional indicators			
Total sedentary time (h/d)	—	—	—
Mean sedentary bout duration (min/d)	0.53	—	—
Total number of sedentary bouts (number of times a day)	−0.63	−0.84	—
Current indicators			
Total sedentary time 30 (h/d)	0.63	0.85	−0.89
Total sedentary time U30 (h/d)	−0.21	−0.76	0.75
Total sedentary time 10 (h/d)	0.83	0.81	−0.93
Mean sedentary bout duration 30 (min/d)	0.08	0.31	−0.21
Secondary indicators			
Total Sedentary time U10 (h/d)	−0.47	−0.83	0.94
Mean sedentary bout duration 10 (min/d)	0.21	0.7	−0.54

The correlation coefficient between *Mean sedentary bout duration* and *Total number of sedentary bouts* is −.84. Furthermore, the correlation coefficients between *Mean sedentary bout duration 10* and *30* and *Total number of sedentary bouts* are −.54 and −.21, respectively (Table 2).

### 3.3 Reliability of indicators

Table 3 shows the ICC_1_ of 4 work days. The ICC_1_ values of all indicators were over 0.8 except for *Total sedentary time 30* and *Mean sedentary bout duration 30* in the 4 work days (Table 3).

**Table 3:** Intraclass correlation coefficients (ICC_1_) in 4 work days

Indicators	ICC_1_ (95% CI)
Conventional indicators	
Total sedentary time (h/d)	**0.83 (0.74‐0.90)**
Mean sedentary bout duration (min/d)	**0.87 (0.77‐0.91)**
Total number of sedentary bouts (number of times a day)	**0.80 (0.69‐0.88)**
Current indicators	
Total sedentary time 30 (h/d)	0.78 (0.65‐0.87)
Total sedentary time U30 (h/d)	**0.80 (0.70‐0.88)**
Total sedentary time 10 (h/d)	**0.81** **(0.70‐0.88)**
Mean sedentary bout duration 30 (min/d)	0.59 (0.35‐0.75)
Secondary indicators	
Total sedentary time U10 (h/d)	**0.82 (0.72‐0.90)**
Mean sedentary bout duration 10 (min/d)	**0.81** **(0.69‐0.88)**

Note

Boldface indicates values of ICC_1_ greater than 0.8.

### 3.4 Criterion validity

Table 4 shows the results of ANCOVA. No significant differences were observed in *Total sedentary time*, *Total sedentary time 10*, *Mean sedentary bout duration 30* among the three BMI groups. Significant differences were observed in *Mean sedentary bout duration*, *Mean sedentary bout duration 10*, *Total number of sedentary bouts*, *Total sedentary time 30*, *U30*, and *U10*. Except for *Mean sedentary bout duration 10*, these indicators showed significant differences between the Underweight and the Normal, or the Underweight and the Overweight groups according to multiple comparison analysis. *Total sedentary time* had very low statistical power of 0.14, while *Total sedentary time U10* and *U30* had sufficient power values ranged from 0.76 to 0.82 (Table 4).

**Table 4:** Results of analysis of covariance (ANCOVA) among the three BMI groups

Indicators	Results of ANCOVA^a^		Adjusted mean (SE)^b^			Multiple comparison analysis^c^
	*P*‐value	Statistical power	Underweight (n = 5)	Normal (n = 31)	Overweight (n = 10)	
Conventional indicators						
Total sedentary time (h/d)	.56	0.14	14.4 (0.5)	14.0 (0.2)	14.3 (0.3)	NS
Mean sedentary bout duration (min/d)	.04*	0.64	20.4 (2.6)	13.4 (1.1)	12.5 (1.9)	U vs O
Total number of sedentary bouts (number of times a day)	.02*	0.70	45.3 (11.2)	78.4 (4.5)	81.1 (8.1)	U vs N, U vs O
Current indicators						
Total sedentary time 30 (h/d)	.03*	0.67	10.3 (0.9)	7.7 (0.4)	7.5 (0.7)	U vs N, U vs O
Total sedentary time U30 (h/d)	.01*	**0.82**	4.2 (0.7)	6.4 (0.3)	6.9 (0.5)	U vs N, U vs O
Total sedentary time 10 (h/d)	.14	0.40	12.7 (0.8)	11.0 (0.3)	10.8 (0.6)	NS
Mean sedentary bout duration 30 (min/d)	.52	0.15	82.7 (9.7)	72.5 (3.9)	69.4 (7.1)	NS
Secondary indicators						
Total sedentary time U10 (h/d)	.02*	0.76	1.5 (0.5)	3.1 (0.2)	3.4 (0.4)	U vs N, U vs O
Mean sedentary bout duration 10 (min/d)	.04*	0.63	50.4 (4.5)	38.1 (1.9)	36.7 (3.3)	NS

a The results of ANCOVA (*P* ‐value and statistical power) among the three BMI groups adjusted for age, sex, and physical activity. Boldface indicates values of statistical power greater than 0.8.

b Adjusted mean of ANCOVA.

c The results of multiple comparison analysis using Bonferroni‐corrected, U: Underweight, N: Normal, O: Overweight, NS: No significant.

* *P* < .05.

## 4 DISCUSSION

This study comprehensively examined the characteristics of indicators of SB, using workers’ objectively measured SB during daily work and life. The indicators were evaluated from the results of each indicator in light of the three perspectives.

### 4.1 Associations between conventional indicators and current indicators

*Total sedentary time U10* and *U30* had a negative correlation with *Total sedentary time*. Our findings for total sedentary time are consistent with some previous studies that showed a negative correlation between total sedentary time and short sedentary bout duration (sedentary bout duration <5 minutes).^8^, ^9^ In addition, our results indicate that *Mean sedentary bout duration* has a strong negative association with *Total number of sedentary bout* that indirectly represents how active the participants took their posture changes between sitting and standing, rather than *Mean sedentary bout duration 10* and *30*.

One study,^23^ indeed, investigating associations between SB and cardio‐metabolic biomarkers reported that the associations disappeared after adjusting total physical activity measured objectively. Other studies in a laboratory setting, furthermore, reported that regularly interrupting SB (increasing the number of short sedentary bouts) was effective to decrease insulin levels and increase energy expenditure.^32^, ^33^, ^34^ These results imply that short sedentary bout duration is not mutually exclusive with physical activity and may be regarded as a state like physical activity rather than that like SB. Previous studies claimed an assumption that both high amounts of SB and prolonged SB increase the risk of health independent of physical activity, and mean sedentary bout duration was often used as the indicator representing prolonged sedentary patterns.^13^, ^21^, ^22^, ^24^ Our results also showed that *Mean sedentary bout duration* and *Total number of sedentary bouts* have an inverse relationship but homogeneous in nature, so that either one might be enough when reporting such statistics. Therefore, to elucidate the independent risk of SB accurately, total sedentary time as the amounts of SB and mean sedentary bout duration as the prolonged sedentary pattern should be calculated excluding less than 10‐minute or 30‐minute bout durations.

### 4.2 Reliability of indicators

Although some studies^35^, ^36^ have addressed that self‐reported sedentary time for workday is more reliable than that for non‐work days, the objective measured indicators using the threshold of 30‐minute such as *Total sedentary time 30* and *Mean sedentary bout duration 30* showed values less than 0.8 of ICC estimates, showing insufficient reliability of measurements within individuals in consecutive 4 work days. This is a notable finding because little has been reported on the reliability of objective measured sedentary time per se within individuals in consecutive working days. The result implies that prolonged sedentary patterns had poor consistencies within individuals in sedentary bout duration of over 30 minutes. Therefore, the more appropriate threshold representing prolonged sedentary patterns might be 10 minutes than 30 minutes.

### 4.3 Criterion validity

In previous studies, it was assumed that SB had a linear association with increasing BMI.^6^, ^7^, ^8^, ^9^, ^37^ Although the results of ANCOVA between three BMI groups showed significant differences in *Mean sedentary bout duration*, *Mean sedentary bout duration 10*, *Total number of sedentary bouts*, *Total sedentary time 30*, *U30* and *U10*, the participants in the Underweight group tended to have high amounts of SB and prolonged sedentary patterns rather than those in the Normal and the Overweight groups. Significant differences between Normal and Overweight were not observed. One of the reasons for this inconsistency with the assumption might be the difference of characteristics of the participants. For instance, participants of a previous study^37^ reporting a significant relationship between prolonged sedentary time similar to *Total sedentary time 30* and BMI, had higher BMI (33.6 ± 5.5 kg/m^2^) than those of the present study (22.0 ± 3.6 kg/m^2^). These results imply that the relationship between SB and BMI might not have a characteristic with a monotonous liner dose‐response relationship.

Another notable finding can be found in the statistical power shown in conventional and current indicators. The statistical power is calculated from sample size, alpha error, and effect size. Cohen 38 claimed that researchers must set desired statistical power values as well as desired significance criteria on the basis of the consideration seriousness of the consequences of the two kinds of errors (alpha and beta errors) and the cost of obtaining data. Moreover when researcher has no other basis for setting the desired statistical power value, he proposed 0.80 as a convention. The present study noted out that *Total sedentary time*, one of the well‐used indicators in previous studies, has very low statistical power of 0.14, while *Total sedentary time U10* and *U30* had sufficient power values ranged from 0.76 to 0.82. Statistical power values requiring larger than 0.8, in general, would demand excessive sample sizes with much effort or at much cost, especially in case of behavioral measurements. Current indicators being used with a 10‐minute or 30‐minute thresholds would be adequate and reasonable as *Total sedentary time* might be inefficient.

As for the calculation of descriptive statistics for each indicator, *Total sedentary time*, *Mean sedentary bout duration*, *Mean sedentary bout duration 10*, and *30* did not follow the normal distribution. Therefore, these indicators might need to be checked the normality carefully and performed log‐transformation prior to analysis, if applicable.

### 4.4 Strengths and limitations of the study

The first strength of this study is scalability due to the ease of measuring SB using the smartphone app. Smartphones are now widespread so that large scale low‐cost research could be made without specific wearable devices. This would be important from the perspective of assessing the risk of SB in epidemiological study fields.

The second point is the representativeness of the data under real work and life scene, which could contribute to reduce of selection bias in the sampling process. Measuring with specific devices could cause selection bias of participants and the healthy worker effect. In addition, participants might be conscious of the device during measurement. In contrast, a smartphone many people use every day has an advantage that the quality of the data could be guaranteed with respect to selection bias and representativeness of daily life.

Finally, non‐measurement times were excluded by using the actual acceleration sensor data and participants’ self‐reports. Some previous studies estimated non‐measurement times like hours of sleep by using only an accelerometer sensor data, so the exclusion of non‐measurement times might have been inaccurate.^7^, ^8^, ^15^ The present study used not only an accelerometer, but also participants’ self‐reports, so the time of exclusion might be more accurate, especially hours of sleep.

Some limitations of the present study should be considered. First, criterion validity was evaluated only with the Japanese workers’ BMI levels. In general, criterion validity may be different depending on health outcomes and the characteristics of participants, such as country, age and BMI. Self‐reported data on individual's height and weight are often used in epidemiological studies though, a recent study in US pointed out that individuals tend to overestimate their height and underestimate their weight. Consequently, BMI based on the self‐reported height and weight might tend to be underestimated. Self‐reported BMI, furthermore, might tend to be affected by characteristics of participants such as some socioeconomic and health‐related factors.^39^

Second, we examined current indicators being used with a 10‐minute or 30‐minute thresholds. Therefore, these thresholds could be insufficient to ensure a no‐monotonic dose‐response, which means that the minimum bout duration defined as SB is unclear. In a future study, the time spent in SB that affects health needs to be examined.

Finally, a step‐counting sensor of a smartphone was used to measure SB, whereas many previous studies used the specific devices using “counts per minute” as the threshold of SB (eg, <100 counts per minute). Thus, the strict definition of SB is not equal between them, the data compatibility with respect to measurement of SB is not guaranteed between a step‐counting sensor of a smartphone and the specific wearable devices. At this time, we did not collect the data to validate criterion validity of measurements between smartphone devices and other specific devices. Therefore, it should be carefully interpreted when comparing the SB related data measured with specific devices to that measured using smartphone sensors. In addition, the data could be measured while participants placed their phones in a chest pocket, but some non‐measurable situations such as while charging or placing in their bags, especially women, might be occurred. It is important to confirm their adherence whether participants wore their own smartphone in their chest pocket for 7 consecutive days except while in bed. Because this might lead to miss‐classification of SB and overestimation of total amount of sedentary time. The next version of the App, upcoming the MotionLogger will implement a self‐report adherence item and an algorithm to detect these situations, using information on gravity sensors.

## 5 CONCLUSION

This study is the first to comprehensively identify the characteristics of current and conventional indicators of SB in light of the association among the indicators, reliability and validity, using objective measurements. The results revealed the rationale of advantage in the current indicator being used with a 10‐minute threshold, rather than the conventional total amount of SB, and also 30‐minute threshold might be available but have some limitations of insufficient reliability of measurements within individuals among consecutive work days.

Further research is required to consider the minimum bout duration defined as SB and use indicators properly according to each purpose of specific studies.

## DISCLOSURE

*Approval of the research protocol:* This study was approved by the Institutional Review Board of Nagoya City University Graduate School of Medical Sciences (No. 60 160 123). *Informed Consent:* Written informed consent was obtained from all participants in this study. *Registry and the Registration No. of the study/Trial:* N/A. *Animal Studies:* N/A. *Conflict of Interest:* The authors declare no Conflict of Interests for this study.

## AUTHOR CONTRIBUTIONS

TE, FM, TM, and NY designed and developed the study protocol and the measurement method. KY analyzed the data and wrote the first draft of the manuscript. KI, TK, SY, and TM supported the analysis and interpreted the data. MK and TE supervised KY with respect to ergonomics and occupational health. TE was responsible for the study as PI and assisted with the drafting of the manuscript. All the authors interpreted the data, contributed to the writing of the manuscript, revised it critically for important intellectual content, and agreed with the final version and the findings.

## References

[1] Biswas A , Oh PI , Faulkner GE , et al. Sedentary time and its association with risk for disease incidence, mortality, and hospitalization in adults: a systematic review and meta‐analysis. Ann Intern Med. 2015;162(2):123‐132. 25599350 10.7326/M14-1651

[2] Healy GN , Matthews CE , Dunstan DW , Winkler EA , Owen N . Sedentary time and cardio‐metabolic biomarkers in US adults: NHANES 2003–06. Eur Heart J. 2011;32(5):590‐597. 21224291 10.1093/eurheartj/ehq451 PMC3634159

[3] George ES , Rosenkranz RR , Kolt GS . Chronic disease and sitting time in middle‐aged Australian males: findings from the 45 and Up Study. Int J Behav Nutr Phys Act. 2013;10:20. 23394382 10.1186/1479-5868-10-20 PMC3571940

[4] Hawkins M , Newman AB , Madero M , et al. The associations of physical activity and television watching with change in kidney function in older adults. J Phys Act Health. 2015;12(4):561‐568. 24762526 10.1123/jpah.2013-0289 PMC4816441

[5] Koster A , Caserotti P , Patel KV , et al. Association of sedentary time with mortality independent of moderate to vigorous physical activity. PLoS ONE. 2012;7(6):e37696. 22719846 10.1371/journal.pone.0037696 PMC3374810

[6] Bellettiere J , Winkler E , Chastin S , et al. Associations of sitting accumulation patterns with cardio‐metabolic risk biomarkers in Australian adults. PLoS ONE. 2017;12(6):e0180119. 28662164 10.1371/journal.pone.0180119 PMC5491133

[7] Celis‐Morales CA , Perez‐Bravo F , Ibanez L , Salas C , Bailey ME , Gill JM . Objective vs. self‐reported physical activity and sedentary time: effects of measurement method on relationships with risk biomarkers. PLoS ONE. 2012;7(5):e36345. 22590532 10.1371/journal.pone.0036345 PMC3348936

[8] Henson J , Yates T , Biddle S , et al. Associations of objectively measured sedentary behaviour and physical activity with markers of cardiometabolic health. Diabetologia. 2013;56(5):1012‐1020. 23456209 10.1007/s00125-013-2845-9

[9] Kim Y , Welk GJ , Braun SI , Kang M . Extracting objective estimates of sedentary behavior from accelerometer data: measurement considerations for surveillance and research applications. PLoS ONE. 2015;10(2):e0118078. 25658473 10.1371/journal.pone.0118078 PMC4319840

[10] Honda T , Chen S , Yonemoto K , et al. Sedentary bout durations and metabolic syndrome among working adults: a prospective cohort study. BMC Public Health. 2016;16:888. 27562190 10.1186/s12889-016-3570-3 PMC5000401

[11] Healy GN , Dunstan DW , Salmon J , et al. Breaks in sedentary time: beneficial associations with metabolic risk. Diabetes Care. 2008;31(4):661‐666. 18252901 10.2337/dc07-2046

[12] Bankoski A , Harris TB , McClain JJ , et al. Sedentary activity associated with metabolic syndrome independent of physical activity. Diabetes Care. 2011;34(2):497‐503. 21270206 10.2337/dc10-0987 PMC3024375

[13] van der Berg JD , Stehouwer C , Bosma H , et al. Associations of total amount and patterns of sedentary behaviour with type 2 diabetes and the metabolic syndrome: The Maastricht Study. Diabetologia. 2016;59(4):709‐718. 26831300 10.1007/s00125-015-3861-8 PMC4779127

[14] Carson V , Janssen I . Volume, patterns, and types of sedentary behavior and cardio‐metabolic health in children and adolescents: a cross‐sectional study. BMC Public Health. 2011;11:274. 21542910 10.1186/1471-2458-11-274 PMC3112118

[15] King WC , Chen J‐Y , Courcoulas AP , et al. Objectively‐measured sedentary time and cardiometabolic health in adults with severe obesity. Prev Med. 2016;84:12‐18. 26724517 10.1016/j.ypmed.2015.12.007 PMC4758881

[16] Hekler EB , Buman MP , Grieco L , et al. Validation of physical activity tracking via android smartphones compared to ActiGraph accelerometer: laboratory‐based and free‐living validation studies. JMIR mHealth uHealth. 2015;3(2):e36‐e36. 25881662 10.2196/mhealth.3505 PMC4414958

[17] Intille SS , Lester J , Sallis JF , Duncan G . New horizons in sensor development. Med Sci Sports Exerc. 2012;44(1 Suppl. 1):S24‐S31. 22157771 10.1249/MSS.0b013e3182399c7d PMC3245518

[18] Ceron J , López D , Ramirez GA . A mobile system for sedentary behaviors classification based on accelerometer and location data. Comput Indus. 2017;92:25‐31.

[19] Hurt CP , Lein DH , Smith CR , et al. Assessing a novel way to measure step count while walking using a custom mobile phone application. PLoS ONE. 2018;13(11):e0206828. 30399162 10.1371/journal.pone.0206828 PMC6219786

[20] Ebara T , Azuma R , Shoji N , et al. Reliability of smartphone‐based gait measurements for quantification of physical activity/inactivity levels. J Occup Health. 2017;59(6):506‐512. 28835575 10.1539/joh.17-0101-OA PMC5721272

[21] Manns P , Ezeugwu V , Armijo‐Olivo S , Vallance J , Healy GN . Accelerometer‐derived pattern of sedentary and physical activity time in persons with mobility disability: national health and nutrition examination survey 2003 to 2006. J Am Geriatr Soc. 2015;63(7):1314‐1323. 26173621 10.1111/jgs.13490

[22] Ezeugwu V , Klaren RE , A. Hubbard E , Manns PT , Motl RW . Mobility disability and the pattern of accelerometer‐derived sedentary and physical activity behaviors in people with multiple sclerosis. Prev Med Rep. 2015;2:241‐246. 26844077 10.1016/j.pmedr.2015.03.007 PMC4721432

[23] Maher C , Olds T , Mire E , Katzmarzyk PT . Reconsidering the sedentary behaviour paradigm. PLoS ONE. 2014;9(1):e86403. 24454968 10.1371/journal.pone.0086403 PMC3893290

[24] Diaz KM , Howard VJ , Hutto B , et al. Patterns of sedentary behavior and mortality in U.S. middle‐aged and older adults: a national cohort study. Ann Intern Med. 2017;167(7):465‐475. 28892811 10.7326/M17-0212 PMC5961729

[25] Martens R , van der Berg JD , Stehouwer C , et al. Amount and pattern of physical activity and sedentary behavior are associated with kidney function and kidney damage: The Maastricht Study. PLoS ONE. 2018;13(4):e0195306. 29617428 10.1371/journal.pone.0195306 PMC5884554

[26] Brakenridge CL , Fjeldsoe BS , Young DC , et al. Evaluating the effectiveness of organisational‐level strategies with or without an activity tracker to reduce office workers' sitting time: a cluster‐randomised trial. Int J Behav Nutr Phys Act. 2016;13(1):115. 27814738 10.1186/s12966-016-0441-3 PMC5097432

[27] Arrogi A , Bogaerts AN , Seghers J , et al. Evaluation of stAPP: a smartphone‐based intervention to reduce prolonged sitting among Belgian adults. Health Promot Int. 2017;34(1):16‐27. 10.1093/heapro/dax046 28973149

[28] Willett WC , Howe GR , Kushi LH . Adjustment for total energy intake in epidemiologic studies. Am J Clin Nutr. 1997;65(Suppl. 4):1220S‐1228S; discussion 1229S–1231S. 9094926 10.1093/ajcn/65.4.1220S

[29] Pedersen E , Danquah IH , Petersen CB , Tolstrup JS . Intra‐individual variability in day‐to‐day and month‐to‐month measurements of physical activity and sedentary behaviour at work and in leisure‐time among Danish adults. BMC Public Health. 2016;16(1):1222. 27914468 10.1186/s12889-016-3890-3 PMC5135790

[30] Landis JR , Koch GG . The measurement of observer agreement for categorical data. Biometrics. 1977;33(1):159‐174. 843571

[31] Craig CL , Marshall AL , Sjostrom M , et al. International physical activity questionnaire: 12‐country reliability and validity. Med Sci Sports Exerc. 2003;35(8):1381‐1395. 12900694 10.1249/01.MSS.0000078924.61453.FB

[32] Peddie MC , Bone JL , Rehrer NJ , Skeaff CM , Gray AR , Perry TL . Breaking prolonged sitting reduces postprandial glycemia in healthy, normal‐weight adults: a randomized crossover trial. Am J Clin Nutr. 2013;98(2):358‐366. 23803893 10.3945/ajcn.112.051763

[33] Dunstan DW , Kingwell BA , Larsen R , et al. Breaking up prolonged sitting reduces postprandial glucose and insulin responses. Diabetes Care. 2012;35(5):976‐983. 22374636 10.2337/dc11-1931 PMC3329818

[34] Swartz AM , Squires L , Strath SJ . Energy expenditure of interruptions to sedentary behavior. Int J Behav Nutr Phys Act. 2011;8:69. 21708007 10.1186/1479-5868-8-69 PMC3141617

[35] Clark BK , Sugiyama T , Healy GN , Salmon J , Dunstan DW , Owen N . Validity and reliability of measures of television viewing time and other non‐occupational sedentary behaviour of adults: a review. Obes Rev. 2009;10(1):7‐16. 18631161 10.1111/j.1467-789X.2008.00508.x

[36] Marshall AL , Miller YD , Burton NW , Brown WJ . Measuring total and domain‐specific sitting: a study of reliability and validity. Med Sci Sports Exerc. 2010;42(6):1094‐1102. 19997030 10.1249/MSS.0b013e3181c5ec18

[37] Healy GN , Winkler EA , Brakenridge CL , Reeves MM , Eakin EG . Accelerometer‐derived sedentary and physical activity time in overweight/obese adults with type 2 diabetes: cross‐sectional associations with cardiometabolic biomarkers. PLoS ONE. 2015;10(3):e0119140. 25775249 10.1371/journal.pone.0119140 PMC4361561

[38] Cohen J . Statistical Power Analysis for the Behavioral Sciences, 2nd edn New York, NY: Lawrence Erlbaum Associates 1988:55‐56.

[39] Chau N , Chau K , Mayet A , Baumann M , Legleye S , Falissard B . Self‐reporting and measurement of body mass index in adolescents: refusals and validity, and the possible role of socioeconomic and health‐related factors. BMC Public Health. 2013;13:815. 24011121 10.1186/1471-2458-13-815 PMC3846114

